# Advanced material and approach for metal ions removal from aqueous solutions

**DOI:** 10.1038/srep08992

**Published:** 2015-03-11

**Authors:** Petri A. Turhanen, Jouko J. Vepsäläinen, Sirpa Peräniemi

**Affiliations:** 1 School of Pharmacy,Biocenter Kuopio, University of Eastern Finland, P.O.Box 1627, FI-70211, Kuopio, Finland

## Abstract

A Novel approach to remove metals from aqueous solutions has been developed. The method is based on a resin free, solid, non-toxic, microcrystalline bisphosphonate material, which has very low solubility in water (59 mg/l to ion free Milli-Q water and 13 mg/l to 3.5% NaCl solution). The material has been produced almost quantitatively on a 1 kg scale (it has been prepared also on a pilot scale, ca. 7 kg) and tested successfully for its ability to collect metal cations from different sources, such as ground water and mining process waters. Not only was this material highly efficient at collecting several metal ions out of solution it also proved to be regenerable and reusable over a number of adsorption/desorption, which is crucial for environmental friendliness. This material has several advantages compared to the currently used approaches, such as no need for any precipitation step.

Disposal of industrial waste water is a major environmental issue since these contaminants can ultimately gain access to surface and ground water which may be used for drinking water purposes. Heavy metals are of special concern because of their persistence. Unlike organic contaminants, heavy metals are not biodegradable and tend to accumulate in living organisms. Furthermore many heavy metal ions are known to be toxic or carcinogenic. Thus removal of these toxic heavy metals from wastewater is of crucial importance to protect the human population and the environment. Several heavy metals are particularly important in the treatment of industrial wastewaters i.e. zinc, copper, nickel, mercury, cadmium, lead and chromium^1^,^2^,^3^,^4^. Various methods exist for the removal of harmful metal ions, particularly heavy metals, from liquids and fluids e.g. waste waters^2^. Chemical precipitation and coagulation-flocculation are the most widely used methods for removing pollutants^1^,^5^. Often metals are removed from solutions by increasing the pH of the effluent, converting the soluble metal into an insoluble form (i.e. its hydroxide)^6^. Flotation^7^,^8^, electrolytic reduction^9^, ion exchange^1^,^10^ and membrane technologies^1^,^6^ are also widely used methods. Recently adsorption has been proposed as representing an alternative treatment procedure^1^,^4^,^5^. Naturally occurring low cost adsorbent materials have been studied: e.g. agricultural waste^4^,^11^,^12^,^13^, industrial by-products^12^,^14^, clays^15^,^16^,^17^,^18^, zeolites^19^,^20^ and chemically modified cellulose materials^21^,^22^. In recent years, many alternative solid-phase sorbents (e.g. carbon nanotubes, fullerens, ion imprinted polymers, biosorbents, nanoparticles) have been investigated. These new materials are claimed to be better than the traditional agents having enrichment performance in the extraction of their target analytes^23^,^24^,^25^,^26^,^27^,^28^. While each of these methods has some benefits, there are also disadvantages e.g. high capital, maintenance or operation costs, laborious procedures, limited capacity or slow speed^1^,^5^,^6^,^12^,^22^. In general, the collection procedure should be simple, relatively rapid, quantitative and not very expensive. The procedure should also require minimum sample treatment. One of the fundamental requirements of any metal collector or adsorbent is its ability to be regenerated and reused over a number of adsorption/desorption cycles since this is both economical and environmentally friendly^4^,^22^.

Bisphosphonates (BPs) with a P-C-P backbone are enzymatically and chemically stable analogues of naturally occurring pyrophosphates. During their 60 years lifetime, BPs have been used for several purposes based on their effective metal chelation properties initially as water softeners^29^ but more recently as bone drugs^30^,^31^,^32^. We have developed a novel, straightforward and rapid method for chromium^III^ ion collection from aqueous solutions and tannery effluents using solid bisphosphonates (BPs) which needs no precipitation step^33^. There do not appear to be any reports in the literature of BPs being utilized in metal ion collection without the presence of some additional resin. In addition, as far as we are aware, no other insoluble chemical compounds have been used in this manner. However, commercially available BP ion exchange resins (Diphonix®) have been used for collecting actinides^34^ and transition metals^35^,^36^. Nevertheless, their production costs are at least 10-fold higher than to the newly-synthetized, insoluble BP described here. Another obvious benefit of our method is that there is no requirement for any precipitation step, which is typically needed if soluble complexation agents are used. This novel microcrystalline “green” BP material called here **N10O** is non-toxic, recyclable, almost insoluble in water but is still an excellent collector of a wide variety of metal cations. Here we describe the effectiveness of **N10O** to collect several other metal ions in addition of chromium^III^ from aqueous solutions and the suitability of the developed method to remove metal ions from real drinking water and mining process water samples in comparison with the commercially available Diphonix® resin.

## Results and Discussion

### Properties of N10O

**Figure 1:**
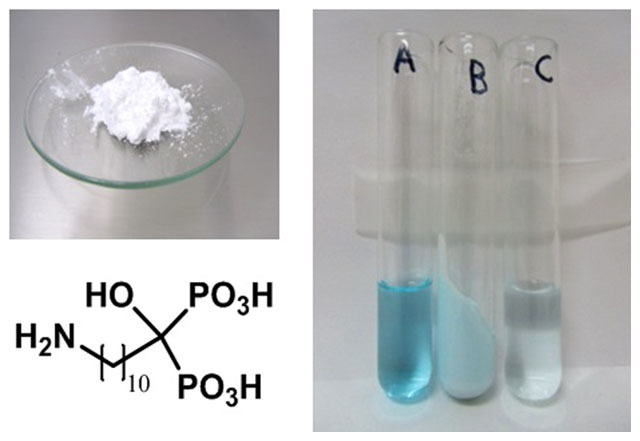
N10O (left picture and chemical structure under the picture) and illustration chelation of Cu^II^ with N10O. (A): 0.1 M CuCl_2_; (B): centrifuged and washed N10O with complexed Cu^II^; (C): solution A after N10O treatment.

Synthesis of 11-amino-1-hydroxyundecylidene-1,1-bisphosphonic acid^37^ (**N10O**, Figure 1) was straightforward at the laboratory scale and also large scale production was successfully demonstrated. The starting materials for the synthesis are economical, which is of crucial importance in commercial terms. The product **N10O** was a fine white porous microcrystalline powder (Figure 1) consisting flake-like crystals (typical dimensions 2 × 30 × 50 μm). Nitrogen BET specific surface area of **N10O** was 11.4 m^2^/g. **N10O** was determined to be insoluble in organic solvents, like chloroform, acetone and dimethyl sulfoxide, since no ^1^H or ^31^P NMR signals were observed in the spectra. In our earlier study^38^ the water solubility of aminobisphospnonates was highly dependent on the carbon chain length in the middle carbon such that with longer carbon chains (n > 7) poor solubilities were obtained. **N10O** is also very sparingly soluble in Milli-Q water (only 59 mg/l at pH 4) and even less soluble in 0.8% and 3.5% NaCl solutions (15 and 13 mg/l, respectively) at pH 6.07.

BPs are known to be good metal chelators due to the superior complexing ability of the O = P-C-P = O moiety, and they have a recognized ability to form metal complexes with relatively high stabilities^39^,^40^. **N10O** also proved to be an efficient metal chelator and the complexation of Cu^II^ to **N10O** was readily evident, since the light blue colour of Cu^II^ in Figure 1, previously present in the solution, was now visible in the solid material leaving behind an almost colourless solution. **N10O** contains a hydroxyl group and two geminally bound phosphonic acid groups (Figure 1), each providing 1–3 donor oxygen atoms to be used as hooks and bridging sites for metal cations. Thus, stable six membered chelate rings could be formed. It is thought unlikely that the amino group participates in the chelation due to its location and zwitterion character. Although **N10O** is sparingly soluble in water and also was almost insoluble during the metal chelation process, it was able to collect effectively metal ions from the solution and they could then be subsequently filtered out of the solution without any additional precipitation step. Moreover, in the case where there was only a single metal in the solution, the chelation process was very rapid (ca. 1 min vortexing and centrifugation), probably due to the large surface area of **N10O**. In addition, the rapid complexation ability was confirmed in a sintered glass crucible experiment (see Experimental section).

In addition, according to the toxicity report (see Supporting info) the material has not exerted any acute toxic effects when this has been examined in rats, Ames-test or in ecotoxicological assays. One of the fundamental requirements of any metal collector or adsorbent is its ability to be regenerated and re-used over a number of adsorption/desorption cycles i.e. it should be both economical in use and environmentally friendly^1^. Based on our simple test on glass crucibles, **N10O** was observed to be regeneratable and reusable at least 20 times (see Experimental section). In fact, the loss of **N10O** in the regeneration steps was even less than expected according to its aqueous solubility determination. This is probably due to the formation of extremely insoluble metal-**N10O** complexes.

### The effect of pH onto the collection of metal ions

The degree of deprotonation of the phosphonic acid groups plays a significant role in complex formation and therefore the pH of the solution is a critical factor in metal collection. The pK_a_-values of the **N10O** could not be determined by a conventional titration method due to the very low solubility of the compound. Previously, we have measured the pK_a_-values for amino-BPs with shorter alkyl chains (pK_1_: 0.60–1.08, pK_2_: 1.70–2.58, pK_3_: 10.04–10.86, pK_4_: 11.65–12.86)^38^ and the pK_a_-values for **N10O** are assumed to be around the same order of magnitude. The OH group bound to the middle carbon is very weakly acidic and does not deprotonate below pH 13^41^. In addition, many metal ions can exist in different forms in solution depending on the pH and this can influence their complex formation with **N10O**.

**Figure 2:**
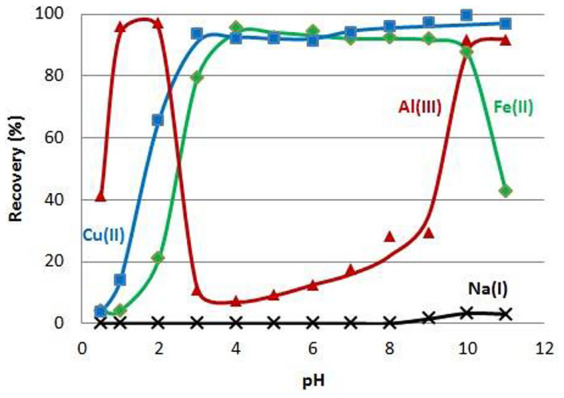
The effect of solution pH onto the metal capture (V = 100 ml, m(N10O) = 100 mg, c(Cu/Fe/Na) = 2 mg/l, c(Al) = 10 mg/l).

The efficacy of collection of several transition metals by **N10O** (Table 1) was studied *via* recovery studies as a function of the pH value of the solution (pH 0.5–11) by the batch method with an excess of complexing agent. Alkali and alkaline earth elements and Al^III^ were also included since they are usually present in sample matrices. The pH graphs of these metal ions were observed to fall into four different classes (Figure 2). For the alkali metal ions, **N10O** proved to be an inefficient collector, since less than 5% of Li^I^, Na^I^, K^I^ and Cs^I^ ions were bound at the mg/l level (see Figure 2 Na(I)). Instead, most of the metal ions (alkaline earth elements, Cr^III^, Fe^III^, Co^II^, Ni^II^, Zn^II^ and Cd^II^) behaved similarly to the Cu^II^ ion: At highly acidic conditions they were not collected, probably because the binding sites on **N10O** are thought to be protonated, resulting in poor metal collection levels. At the optimum pH range, the binding sites are left partially unprotonated and maximal metal binding is possible over a wide range of pH values (Figure 2, Table 1). However, the lower pH limit of efficient collection differs from one metal ion to the next as can be seen from the pH_1/2_ values (pH_1/2_ is the pH value at which 50% of metal ions are collected) in Table 1. This feature could be useful in the separation of metal ions from each other (e.g. Fe^III^ and Cu^II^ could be efficiently collected at lower pH's than the other metals) and the collected metal ions could probably be individually removed from **N10O** by washing it with acid solutions of varying strengths.

Mn^II^ and Fe^II^ ions behave very similarly as the metal ions mentioned above, except that their recovery decreased at pH above 10 (see Figure 2 Fe^II^). Instead, the behaviour of Al^III^ ion differed substantially from the other metal ions studied. Al^III^ ion reveals two high maxima in the pH graph (Figure 2), probably due to its tendency to form an amphoteric hydroxide, and thus it could be collected both at low and elevated pH values. On the whole, further by optimizing the collection procedures (e.g. amount of **N10O**, contact time, stirring) it is anticipated that even more efficient collection of these metal ions could be achieved.

### Metal capacities

One of the most important variables required in the design of adsorption processes for the separation and purification of liquid mixtures is the capacity of the adsorbent for any given component^42^. The ability of **N10O** to collect different metal ions was clarified in these capacity studies. The recoveries of the metal ions were determined by the batch method with an excess of complexing agent at constant pH 4.0 (except Al^III^ at pH 1.0). The **N10O** uptakes of metal ions (Table 2) varied extensively; from 0.05 mol/mol for Fe^III^ to 0.45 mol/mol for Ca^II^. As a comparison, the corresponding capacities (mg/g dry weight, Table 2) for the commercially available Diphonix® resin were determined under the same conditions. Diphonix® resin (**2**, see Experimental section) has a BP moiety (pK_1_ = 1.5, pK_2_ = 2.5, pK_3_ = 7.2, and pK_4_ = 10.5) as a part of the resin material, but it contains also some other functionalities (strongly acidic sulfonic and weakly acidic carboxylic acid groups)^36^. The moisture content of Diphonix® resin is 70%.

The uptakes of Fe^II^ and Al^III^ ions were at the same level for both **N10O** and Diphonix® resin (Table 2). **N10O** exhibited higher uptakes of Mn^II^, Co^II^, Cu^II^, Zn^II^ and Cd^II^, whereas the uptakes of Fe^III^, Cr^III^, Ni^II^ and Sr^II^ were greater with Diphonix® resin. However, in some cases the lower Fe^III^ uptake with **N10O** can even be beneficial *e.g.* when the other metal ion collection is desired in a sample containing a high concentration of Fe^III^ in the matrix^31^. **N10O** displayed higher uptakes of Mg^II^, Ca^II^ and Ba^II^ than to Diphonix® resin which can be disadvantageous in some instances, since Mg^II^ and Ca^II^ are commonly present in different sample matrices. However, in competition situations, these estimates are not so straightforward, since also the stabilities of the complexes need to be taken account. In general, the levels of uptake with **N10O** were comparable with both naturally occurring adsorbent and modified cellulose materials as well as with commercial ion exchange resins^1^.

### Interaction of metal ions

The solutions emitted by industrial processes and wastewaters usually contain more than one metallic species. Therefore, it is essential to investigate the sorption behaviour under competitive conditions i.e., when several metallic species are present. In such multicomponent liquids, the metal complexing capacities alone do not provide an adequate perspective. In fact, the metal ions are competing with each other for complex formation with **N10O** and thus stability constants provide more information for the prediction of multicomponent liquid adsorption equilibria. Unfortunately, the stability constants of the **N10O** metal ion complexes could not be determined by conventional methods due to the very low solubility of **N10O** itself and also of the formed complexes in water and organic solvents. Generally, BPs are known to be good metal ion chelators, forming stable complexes with several metal ions. The stability constants for complex formation (log β) have usually been measured for etidronate^36^,^43^,^44^,^45^,^46^,^47^, pamidronate and alendronate^48^,^49^,^50^ (e.g. Cu^II^ log β 20.1^46^, 29.53^48^ and 30.20^48^, respectively).

In the evaluation of metal ion competition for complex formation with **N10O** in binary systems, batch adsorption tests were conducted employing equal molarities (0.137 mmol) of the two metal ions at a constant pH value. These divalent metal cations which had the highest **N10O** uptakes were selected in the experiments. Mn^II^ ion had to be omitted because it precipitated out at the selected concentration level and pH value (pH = 4.0). Then the solid complexing agent **N10O** (2:1 mol/mol) was added to the binary metal ion solution. Afterwards, the unbounded metal ion concentrations were determined by AAS and the bound metal ion amounts (mol) and bounding ratios (mol/mol) were calculated (Table 3). On the basis of the results the order of bounding was estimated as follows: Cu^II^ > Zn^II^ > Fe^II^ > Cd^II^ > Co^II^ > Mg^II^ > Ca^II^ > Ni^II^. Thus, if the **N10O** amount is limited, Cu^II^ ions would be expected to be most effectively collected in preference over the other ions and even the presence of high Ca^II^ and Mg^II^ concentrations in a sample may not necessarily prevent the ability of **N10O** to bind Cu^II^.

### Ground water samples

There are many studies demonstrating that the natural surface water and groundwater can became contaminated by heavy metals either due to anthropogenic sources or natural geological origin^51^. Conventional treatment systems are not always capable of removing completely the harmful metals in the water especially when they are present at low concentrations^2^, thus alternative purification methods are needed to improve the current treatment process. Well waters from the Finnish countryside were used for testing the ability of function of **N10O** as a metal remover. The ground waters in Finland are mainly soft calcium bicarbonate-containing waters^10^ and the quality of ground waters for household consumption may differ widely from location to location^52^. The high concentrations of iron and manganese, which often appear in the same ground waters, are problematic since they can cause technical problems in water supplies, staining of water and water fixtures^10^,^53^. Recent studies have also suggested, that exposure to manganese from drinking water can pose health concerns, especially in children^54^. In water systems, corrosion of piping and fittings in water delivery systems can release metals (e.g. iron, copper and zinc) in water and also metal-containing sediments occasionally released from pipe surfaces can elevate the concentrations of these metals. Aluminum is generally present at low concentrations in both ground and surface waters, but higher concentrations are likely to exist in more acidic waters because pH has a major impact on its mobility^53^,^54^. In some cases, water hardness can also cause problems (mainly Ca^II^ and Mg^II^)^10^.

Several ground water samples were used for testing with four representative examples being shown in Table 4; three dug wells (WW1–3) and one drilled well (WW4). The measured initial metal concentrations were mostly below the technical guidelines also shown in Table 4, but some values did exceed them (e.g. Al and Fe in WW1 and WW2, Mn in WW1 and WW4). The removal percentages demonstrate that **N10O** effectively collected copper, zinc, manganese and alkaline earth elements from all of the water samples. Aluminum was also effectively removed from WW1, but slightly poorer results were achieved with WW2. It is thought that in this sample, the metals were not completely in the free, ionic form favorable for complexation. Unexpectedly, sodium and potassium were also partially removed, possibly due to co-precipitation. Treatment of water samples with commercial Diphonix® resin achieved similar removal results (results are not shown here), except for zinc (about 10% poorer values). However, when compared to Diphonix® resin, **N10O** is a much less costly material due to its inexpensive starting materials and straightforward production, which makes its use especially attractive. Moreover **N10O** is also easier to handle since it is entirely solid and contains no moisture unlike Diphonix® resin (70% moisture).

The results clearly indicate that **N10O** is capable of removing harmful metals from water even if they are present at low concentrations, and it can also reduce water hardness. However, although **N10O** is nontoxic and its solubility in water is very low, even a small amount of **N10O** remaining in water (on average 35 mg/l in these ground water samples) might restrict its usefulness for drinking water applications, but it still could be efficiently used in certain industrial purposes, e.g. treatment of raw water.

### Mining process water samples

Industrial waste constituents are a major source of several kinds of metal pollution in natural waters. For example, the rapid and often unregulated industrialization in developing countries has led to increase disposal of heavy metal into the environment. In the developed world, there are more and more stringent regulations, which require that the concentrations of heavy metals need to be reduced to safe levels before they can be released into the environment, a serious challenge to many industrial concerns^2^. The ability of **N10O** to remove metal ions from solutions containing higher metal ion concentrations was tested by using process water samples from a mining company. At first, the efficacy of removal of metal ions from two samples MPW1 and MPW2 (Table 5) with different metal ion concentrations was tested with **N10O** and also Diphonix® resin as a comparison.

**Figure 3:**
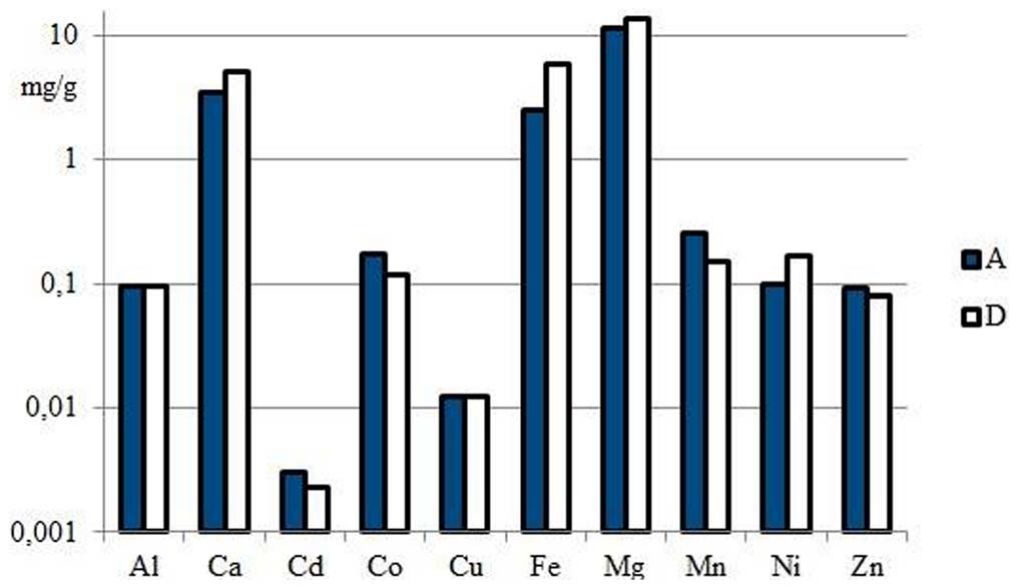
The total amounts of metals (mg/g) removed by N10O (A) or Diphonix® (D) from MWP1 sample (t = 24 h).

In the case of sample MPW1, the results indicate clearly that the amount of adsorbent used (10 g/l **N10O** and 30 g/l Diphonix® resin) were too low for this sample and would need to be increased. Only aluminum and copper ions were effectively removed with both adsorbents (also iron ions in the case of Diphonix® resin), whereas Mn^II^, Co^II^, Ni^II^, Zn^II^, and Cd^II^ ions were poorly collected (Table 5, Figure 3). The high removal per cent of Cu^II^ was not surprising since Cu^II^ ion had been previously observed to compete successfully in complex formation with other metals ions (Table 3). The percent removals of Mn^II^, Zn^II^ and Cd^II^ were higher with **N10O** whereas Diphonix® resin collected Fe^II/III^ and Ni^II^ ions more effectively, as expected on the basis of the capacity experiments. Instead, **N10O** collected less calcium and magnesium ions than Diphonix® resin which was somewhat surprising because **N10O** had higher Ca(II) and Mg(II) capacities (Table 2), but then again calcium and magnesium ions were almost last ones in bounding order (Table 3) and probably they lost the competition for coordination sites. On the whole, both adsorbents removed mainly magnesium, calcium and iron ions (Figure 3) from MPW1.

**Figure 4:**
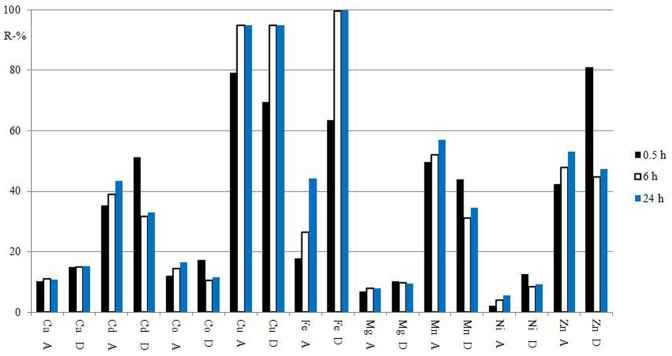
The effect of contact time onto the removal per cents (R-%) of metal ions from MWP1 sample by N10O (A) and Diphonix® (D).

The contact time can have a major impact on the removal of metals ions: the structure of the ligand material (**N10O**) is porous material probably containing different sizes of pores whereas in Diphonix® the resin complexing groups are present on the surface of the resin particles) and sample composition (metal ions and their concentrations) may affect the time needed to reach a state of equilibrium. The effect of contact time on the collection of metal ions was studied with MPW1 sample. With **N10O** (10 g/l) a longer contact time was advantageous, since the removal efficacies systematically increased with increasing contact time regardless of metal ion (Figure 4). This may be due to the porous structure of **N10O** as diffusion of metal ions inside the material requires more time. Instead, with Diphonix® resin, the increase of contact time improved the removal of Mg^II^, Ca^II^, Fe, Al and Cu^II^ ions from sample solution, but meanwhile reduced the removal of Cd^II^, Co^II^, Mn^II^, Ni^II^ and Zn^II^ ions. This behaviour may be a result of competition of metal ions for complexation sites on the surface of resin particles and long contact time may be disadvantageous if the removal of latter ions is desired.

Metal ion concentrations were significantly lower in sample MPW2 and considerably higher removal per cents were achieved (Table 5) thus adsorbent amounts used (10 g/l **N10O** and 30 g/l Diphonix® resin) were more satisfactory for this specimen. The third sample MPW3 contained also very high metal ion concentrations, but increasing amount of **N10O** to 50 g/l achieved excellent removal per cents for metal ions (Table 5), evidence that **N10O** can be used for this kind of samples after optimizing the collection conditions. As before, Na and K ions were only partially removed probably due to co-precipitation. The results above indicate that **N10O** could be utilized for several purposes e.g. removal of harmful metal ions from waste waters or recovery of useful metal ions from mining tailings. In summary, the process must be optimized carefully for the type of sample in order to ensure the removal of the desired metal ions.

## Conclusions

The novel method using solid BP called **N10O** as a metal collector is readily feasible for use in the purification of ground waters and industrial waste waters. This method has many advantages over currently used methods. Firstly, the process is very simple: a solid BP is directly dispersed into a solution containing metal ions which need to be removed. The only sample pretreatment step required is pH adjusting if the pH of sample solution is not appropriate for **N10O** metal collection. Interestingly, metal cations can be effectively collect from real waste water samples into this solid material with good binding capacities only within a few hours without any precipitation step. In addition, to being reusable and non-toxic, **N10O** possesses several other advantages over to similar approaches: ability to capture of many metals simultaneously, the need for minimum sample treatment, high capacity, feasible for use over a wide pH range, possibility for pH selective metal collection, ease of the separation process, inexpensive capital cost, suitable for a wide variety of target pollutants and good recyclable properties. Furthermore, **N10O** has a low affinity for Fe^III^, thus it could be utilized in the removal of other metals from solutions containing high concentrations of Fe^III^. **N10O** has also proven to be effective collector of solutions with high Mg and Ca contents.

Finally, based on initial screening with our commercial partners, **N10O** can be considered as a extremely potential next generation complexation agent suitable for a variety of industrial applications. Typically, unwanted metal cations are not only contaminating process and waste waters, but also occur in waters draining through dump sites, ash from waste burning and in well waters. Metal ions may also be present in the chemicals which are to be used as water purification agents or in paper mills^1^. Another possible application is recovery of valuable metals (e.g. noble metals and rare earth elements) from different kinds of solutions and extracts^55^. Furthermore the development of new solid-phase materials as sorbents and their application in preconcentration methods for the determination of trace elements is also an interesting scientific topic. It is often difficult to make a direct determination of extremely low concentrations of certain elements by routine analytical techniques. The limitations can be associated with matrix interference or insufficient sensitivity of analytical techniques. In such situations, a preliminary separation and a preconcentration step of the trace elements from the matrix are frequently required^24^. With **N10O**, it would be possible to preconcentrate metal ions from the solution onto a compact light solid matrix (e.g. a kind of disc), to be analyzed by either by direct analytical methods (e.g X-ray fluorescence spectrometry), or after subsequent desorption into small volume and detection by other techniques, handling liquid samples.

The material (**1**, **N10O**) is available for research purposes i.e. quatities from 1 g up to 1 kg on request. For commercial purposes (trade name: CH Collector), please contact Oy Chemec Ab (http://www.chemec.fi/en). This material has already been proposed to be as a part of water purification system in recent review article^56^.

## Methods

All tests were performed in three replicates.

### Chemicals, standard solutions and general information

11-Aminoundecanoic acid was purchaced from Acros; phosphorus trichloride, phosphorous acid and methanesulfonic acid were purchased from Sigma-Aldrich. Suprapur® hydrochloric acid 30% was purchased from Merck. Metal stock solutions were all Titrisol standards purchased from Merck, except Fe^II^ solution, which was prepared from FeCl_2_·4H_2_O salt (Merck). All standard solutions were prepared in Milli-Q water. All stock and standard solutions were stored at 4°C until use. Diphonix® resin was purchased from Eichrom.

^1^H, ^31^P and ^13^C NMR spectra were recorded on a Bruker Avance 500 spectrometer operating at 500.1, 202.5 and 125.8 MHz, respectively. TSP was used as an internal standard for ^1^H and ^13^C measurements, and 85% H_3_PO_4_ was used as an external standard for ^31^P measurements. The *^n^J_CP_* couplings were calculated from carbon spectra with the coupling constants given in parenthesis as hertz. Particle size of **N10O was** determined by JEOL JEM-2100F Transmission Electron Microscope and surface area by BET method^57^. Metal concentrations were analyzed either by a Perkin Elmer 5100 atomic absorption spectrometer (AAS) by using air-acetylene flame or by an inductively coupled plasma optical emission spectrometer (ICP-OES). A Thermo Electron iCAP 6600 Duo View equipped with Cetac ASX-520Hs and an autosampler were used. Solubility of **N10O** in water and phosphorus content of **N10O** were determined at 880 nm by Jasco V-530 spectrophotometer using the molybdenum blue method^58^. Liquid samples were filtered (0.2 μm membrane filter) and solid samples were decomposed with nitric acid by the microwave digestion technique using CEM MDS-81D Microwave System prior to determination. Elemental analysis (C, H, N) was accomplished with a ThermoQuest CE Instruments EA 1110-CHNS-O elemental analyzer (CE Instruments, Milan, Italy).

### Synthesis, isolation and purification of solid chelation material, 11-Amino-1-hydroxyundecylidene-1,1-bisphosphonic acid (N10O) (see Figure 5) and its characterization data

**N10O** was synthesized using the method previously reported^59^. The isolation and purification procedures are somewhat different and will be reported here in detail. A mixture of 11-aminoundecanoic acid (157 g), phosphorous acid (64 g), and methanesulfonic acid (375 ml) was heated to 65°C followed by addition of PC1_3_ (140 ml) for over 1–2 hours. The mixture was maintained at 65°C for 48 h and cold distilled water (1 L) was added to a cooled solution with vigorous stirring. After refluxing overnight, the reaction mixture was allowed to cool to room temperature (r.t.) (usually overnight) and the solid product was collected by filtration. Filtered crude product was added to a 2 L flask and distilled water (1 L) was added and the mixture was heated to approx. 70–80°C with vigorous stirring. After the mixture was cooled to ca. 50°C, white solid was filtered, washed with 1 M HCl (approx. 700 ml), distilled water (ca. 1.5 L) and finally with acetone (0.5 L). Final product (**1**) was allowed to dry in r.t. approx. 48 h before it was obtained as white powder (271 g, 95% yield) When prepared in approx. 1 kg scale (four times of all amounts reported above) yield was 90%. **N10O** has also been prepared successfully in pilot scale (approx. 7 kg batch, but the procedure is not reported here). ^1^H NMR (D_2_O + 1 drop of 6 M NaOD, 500 MHz) δ 2.60 (t, 2H, ^3^*J*_HH_ = 7.5), 1.93–1.82 (m, 2H), 1.60–1.51 (m, 2H), 1.47–1.39 (m, 2H), 1.36–1.23 (m, 12H). ^13^C NMR (D_2_O + 1 drop of 6 M NaOD, 500 MHz) δ 79.6 (t, ^1^*J*_CP_ = 134.2), 43.4, 39.0, 34.5, 33.3, 31.95, 31.94, 31.6, 31.4, 28.9, 27.2 (t, ^2^*J*_CP_ = 5.3). ^31^P NMR (D_2_O + 1 drop of 6 M NaOD, 202 MHz) 20.4. Anal. Calcd. for C_11_H_27_O_7_P_2_N·H_2_O: C, 36.17; H, 8.00; N, 3.83; P, 16.96. Found: C, 36.20; H, 8.03; N, 3.73; P, 16.94.

### Solubility determination

Solubility of **N10O** in aqueous solution was determined at constant room temperature (21.0°C) by preparing a saturated solution in Milli-Q water without buffering. The mixture containing an excess of **N10O** was first agitated for 30 min with a magnetic stirrer and then the sample mixture was allowed to stand for 24 h without stirring. A sample (ca. 5 ml) was drawn from the liquid above the solids, pH was measured and the sample was filtered through a 0.2 μm membrane filter to prevent the presence of possible insoluble particles. The phosphorus concentration in the filtered sample solution was determined with a spectrophotometer as described above and it was used to calculate the solubility of the compound. Solubility experiments of **N10O** in 0.8% and 3.5% NaCl solutions at pH 6.07 were performed similarly as described above. Instead, the solubility of **N10O** in tap waters (in Chapter 2.5) was calculated on the basis of the difference of the phosphorus concentrations in untreated and **N10O** treated samples.

### Test tube experiment

White solid **N10O** (1.3 g) was vortexed with 0.1 M CuCl_2_ solution (7 ml) in a glass tube for 1 min. before centrifugation. The solution lying above the solids was removed to another glass tube and observed to be almost colourless and the solids (pale blue) were washed with water, vortexed for 1 min. and centrifugated (repeated twice). Solids were observed to maintain its pale blue colour.

### Sintered glass crucible experiment

White solid **N10O** (3.0 g) was placed in a sintered glass crucible (G4) and 0.1 M CuCl_2_ solution (35 ml) was poured on top of it which the passed slowly through the **N10O** with vacuum suction. The **N10O** layer in the sintered glass had turned from white to pale blue and the colour remained even when washed with a large amount of distilled water indicating the formation of insoluble **N10O** Cu^II^ complex. Cu^II^ was liberated from the complex by addition of 1.0 M HCl through the complex in the sintered glass, and after this the colour of **N10O** changed from pale blue to white again and the HCl solution which had been in contact with **N10O** changed from being colourless to blue.

### Regeneration experiment

Regeneration experiments were conducted by sucking alternatively 20 ml 0.1 M CuCl_2_ solution and 25 ml 0.5 M, 1.0 M or 2.0 M HCl solutions through **N10O** (0.700 g) in glass crucibles (G4). Regeneration could be achieved on twenty successive occasions without any significant loss of Cu^II^ uptake efficacies. HCl effectively removed Cu^II^ from **N10O**, even in 0.5 and 1 M concentrations and no notable loss of **N10O** due to increased solubility was observed. Instead, with 2.0 M HCl approx. 10% of **N10O** was lost over the 20 regeneration procedures.

### pH vs Recovery experiment

The recoveries of metal ions were determined as a function of pH by the batch method in the presence of an excess of **N10O**. The sample pH was adjusted with HCl or NaOH solutions of appropriate concentrations and initial metal ion concentration was measured by atomic absorption spectrometry (AAS) after filtration (0.2 μm membrane filter). **N10O** (m = 100 mg) was added to the sample (V = 100 ml) and mixture was agitated 24 h with a magnetic stirrer. After filtration, the final metal ion concentration was measured again. The recovery per cent of metal ions was calculated from the initial and final metal ion concentrations.

### Capacity experiment

**Figure 6:**
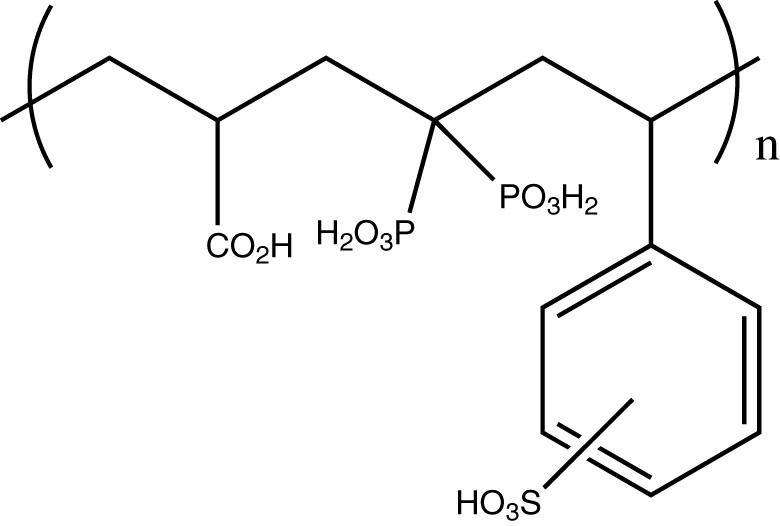
Chemical structure of Diphonix®.

The metal complexing capacities for **N10O** and Diphonix® (see chemical structure in Figure 6) were determined by batch method in the excess of metal ion (C(M^n+^) = 100 mg/l) at pH 4.0, (except Al^III^ and Fe^III^ pH 1.0 and 3.0, respectively). The amount of **N10O** used was 100 mg and Diphonix® resin 300 mg, because moisture content of Diphonix® resin is 70%. Experiments were accomplished otherwise as described above. Uptake of metal ion per unit mass of **N10O** or Diphonix® resin (mg/g) was calculated as q = (C_0_-C) ·V/W where C_0_ and C (mg/l) corresponded to liquid-phase concentration of metal ion initial and final sampling times, respectively, V is the volume of solution (l), and W is the mass of dry **N10O** or Diphonix® resin used (g).

### Interaction experiments

In interaction experiments to the synthetic binary solution (V = 0.100 l) of metal ions of equal molarities (C(M^n+^) = 1.37 mM) complexing agent **N10O** (m = 0.100 g, n = 0.274 mmol) was added. Otherwise samples were treated in a similar way as in the recovery experiments described above. Metal concentrations were measured by AAS and metal amounts (mol) bounded were calculated on the basis of the difference of initial and final metal concentrations and bounding ratio (mol/mol) was calculated for binary system.

### Experiment with ground water samples

Ground water samples (WW1–4) were taken from the tap after water was led into the building. Before the sampling, the water was allowed to run for several minutes. The pH values of samples were measured (waters 1–4 pH 7.11, 7.37, 8.06 and 8.50, respectively) but not readjusted prior to the addition of either **N10O** or Diphonix® and the initial metal concentrations (see Table 2) were measured with an inductively coupled atomic emission spectrometer (ICP-OES). **N10O** or Diphonix® (0.100 g or 0.300 g, respectively) was added to the water sample (100 ml) and the mixture was agitated with a magnetic stirrer 24 h. After filtration (0.2 μm membrane filter) metal ion concentration was measured again. Removal per cents of metal ions from well waters were calculated by determining as the final metal ion concentrations divided by their initial pretreatment concentrations.

### Experiment with mining process water samples

Mining process water samples (MPW 1 and 2) were taken directly from two different process locations. The pH values of samples were 4.55 and 5.12, respectively. Otherwise samples (100 ml) were treated and the results calculated in a similar way as in the case of well waters, except that the amounts of **N10O** or Diphonix® were greater (1.000 g or 3.000 g, respectively). For sample, MPW 3 an even greater amount of **N10O** was used (5.000 g). The contact time experiments for sample MWP1were conducted with **N10O** (10 g/l) in the same way as above except that the contact times were 0.5 h, 6 h or 24 h.

## Author Contributions

P.T. has synthesized the N10O material and was the first one to observed its potential. S.P. performed most of the experiments. S.P. and J.V. planned the most of the performed experiments. The manuscript was written by S.P. and P.T. All authors reviewed the manuscript.

## Supplementary Material

Supplementary Information Supporting Info

## Figures and Tables

**Figure 5:**
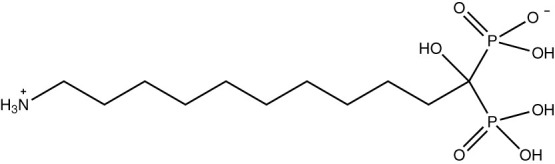
Chemical structure of N10O (zwitterionic form).

**Table 1:** The pH_1/2_-values and the optimum pH-ranges for the collection of single metal ions and the minimum recoveries at optimum pH-range (V = 100 ml, m(N10O) = 100 mg)

Ion	c (M) (mg/l)	pH_1/2_[a]	Optimum pH range	Recovery (%)
Mg^II^	0.5	3.7	4–11	>74
Ca^II^	2.0	3.6	4–11	>50
Sr^II^	2.0	3.6	4–11	>82
Ba^II^	10	2.6	3–11	>70
Cr^III^	4.0	2.5	3–11	>94
Mn^II^	1.0	3.5	5–10	>82
Fe^II^	1.0	2.5	3–10	>80
Fe^III^	1.0	0.8	2–11	>72
Co^II^	2.0	3.3	4–11	>77
Ni^II^	2.0	4.4	4–11	>40
Cu^II^	2.0	1.6	3–11	>87
Zn^II^	0.5	3.4	4–11	>72
Cd^II^	0.5	3.3	4–11	>70
Al^III^	10		1–2, 10–11	>91

^[a]^pH_1/2_ = pH value at which 50% metal ion is collected.

**Table 2:** Uptakes of single metal ions for **N10O** and Diphonix® with an excess of metal ion by the batch method (V = 100 ml, m(N10O) = 100 mg, m(Diphonix) 300 mg)

		**N10O**	**N10O**	Diphonix®
Ion	pH	Uptake (mol/mol)	Uptake (mg/g)	Uptake (mg/g)
Mg^II^	4.0	0.38	25.6	16.3
Ca^II^	4.0	0.45	49.4	23.6
Sr^II^	4.0	0.08	19.6	30.8
Ba^II^	4.0	0.21	78.5	30.6
Cr^III^	4.0	0.03	4.9	22.6
Mn^II^	4.0	0.07	9.8	3.2
Fe^II^	4.0	0.27	41.9	46.0
Fe^III^	4.0	0.05	7.5	35.0
Co^II^	4.0	0.27	43.2	28.5
Ni^II^	4.0	0.08	12.6	31.6
Cu^II^	4.0	0.35	58.0	29.5
Zn^II^	4.0	0.40	68.6	26.2
Cd^II^	4.0	0.24	71.3	50.9
Al^III^	1.0	0.14	10.2	11.7

**Table 3:** The effect of metal ions on the removal of metal ions other by **N10O**. (n(M^2+^) = 0.137 mmol, n(**N10O**) = 0.274 mmol, V = 100 ml, pH = 4.0)

	Bounding ratio (mol/mol)						
X	X/Ni^II^	X/Ca^II^	X/Mg^II^	X/Co^II^	X/Cd^II^	X/Fe^II^	X/Zn^II^
Cu^II^	16.2	42.7	22.3	2.1	7.7	1.8	4.0
Zn^II^	9.2	5.7	4.4	4.1	2.6	1.2	
Fe^II^	397	8.1	6.8	2.3	2.5		
Cd^II^	3.3	6.7	2.0	2.8			
Co^II^	12.8	2.5	2.1				
Mg^II^	4.7	1.4					
Ca^II^	2.5						

**Table 4:** Initial metal concentrations in well waters (WW1–4), removal percentages (R) of metal ions by **N10O** treatment (V = 100 ml, m(N10O) = 100 mg) and technical guidelines for drinking water quality

	WW1		WW2		WW3		WW4		
Ion	c (mg/l)	R (%)	c (mg/l)	R (%)	c (mg/l)	R (%)	c (mg/l)	R (%)	Technical guidelines^54^ (mg/l)
Na^I^	6.7	43.0	21.7	26.3	16.0	35.6	12.2	31.9	150
K^I^	12.0	61.6	7.4	46.8	2.4	76.2	11.5	49.7	12
Mg^II^	1.9	>95	16.9	89.2	5.4	>98	8.2	>99	50
Ca^II^	13.9	99.0	24.4	98.7	24.5	100	21.6	99.5	100
Sr^II^	0.09	>79	0.12	>83	0.09	>78	0.12	>83	Nd
Ba^II[a]^	0.09	>77	0.21	>91	<DL		0.08	>76	Nd
Al(tot.)^[b]^	6.0	79.6	0.3	48.3	<DL		<DL		0.2
Mn(tot.)^[a]^	0.07	>70	0.05	>57	<DL		0.48	>96	0.05
Fe(tot.)^[c]^	6.1	>97	0.7	40.1	<DL		<DL		0.2
Cu^II[b]^	<DL		0.97	>95	<DL		<DL		1.0
Zn^II[a]^	0.03	>31	0.61	>97	<DL		0.03	>40	3.0

> = final metal concentration below detection limit (DL) and DL used in calculations, Nd = not defined, DL (mg/l) = [a] 0.02, [b] 0.05, [c] 0.1.

**Table 5:** Initial metal concentrations in mining process waters (MPW(1–3)) and removal percentages of metal ions by **N10O** and Diphonix® resin treatment (t = 24 h)

	MPW1			MPW2			MPW3	
		Removal-%			Removal-%			Removal-%
Ion	c (mg/l)	**N10O**	Diphonix	c (mg/l)	**N10O**	Diphonix	c (mg/l)	**N10O**
Na^I^	123.7	1.3	0	9.4	0	0	158.0	54.4
K^I^	97.2	0.6	2.0	9.4	13.0	0	808.0	74.4
Mg^II^	1426	7.9	9.5	151	82.2	96.7	389.0	99.8
Ca^II^	327	10.7	15.3	32.2	87.7	97.8	23.5	99.1
Al(tot.)^[a]^	1.13	>84	>84	<DL	-	-	29.6	99.7
Cr^III[b]^	<DL	-	-	<DL	-	-	2.78	99.6
Mn(tot.)	4.44	57.0	34.2	0.47	>97	>97	11.6	99.8
Fe(tot.)	57.6	44.2	>99	6.6	>99	>99	8.5	71.8
Co^II[b]^	10.4	16.5	11.4	1.3	92.8	>99	<DL	-
Ni^II^	18.4	5.4	9.1	2.6	57.6	99.4	0.16	>88
Cu^II[a]^	0.13	>95	>95	<DL	-	-	<DL	-
Zn^II^	1.70	53.1	47.2	0.19	>93	>93	83.0	100
Cd^II[c]^	0.069	43.7	33.0	0.008	>86	>86	<DL	-

> = final metal concentration below detection limit (DL) and DL used in calculations, - = initial metal concentration below DL, DL (mg/l) = [a] 0.05, [b] 0.02, [c] 0.01, liquid to solid ratio MPW1 and MPW2 100:1 both A and D, MPW 100:5.
