# Supplementary material for: Advanced material and approach for metal ions removal from aqueous solutions
Source: Sci Rep. 2015 Mar 11;5:8992. doi: 10.1038/srep08992 (PMC4355739; doi:10.1038/srep08992)
Supplement: Supplementary Information — Supporting Info [file srep08992-s1.pdf]

Supporting Information

Advanced material and approach for metal ions removal from aqueous solutions

Petri A. Turhanen*, Jouko J. Vepsäläinen and Sirpa Peräniemi

School of Pharmacy, Biocenter Kuopio, University of Eastern Finland, P.O.Box 1627, FI-70211, Kuopio, Finland. E-mail: petri.turhanen@uef.fi

SUMMARY STUDY REPORT

This document here is shortened by Jouko Vepsäläinen from the original report.

Summary on the Estimation of Potential Toxicity of the 11-amino-1-hydroxyundecylidene-1,1,-bisphosphonic acid

Date: 31.12.2010

Author or the original report (ca. 70 pages):
Mari Madetoja, M.Sc.
European Registered Toxicologist

Infodix Discovery Informatics / Biodix Discovery Biology / Toxis In vivo Services / novamass ADME/DMPK / Medachem Medicinal Chemistry / 4Pharma Clinical Services

SBW Corp. Ltd | Spektri Business Park, Metsanneidonkuja 6, 02130 Espoo, Finland | Phone: + 358 - 10 439 9440 | Fax: +358-9-678 783 | www.sbw.fi

1. Material

Test Item 11-amino-1-hydroxyundecylidene-1,1,-bisphosphonic acid

	Test item
Name	11-amino-1-hydroxyundecylidene-1,1,-bisphosphonic acid
Manufacturer/Sponsor	University of Eastern Finland
Batch number	PT/MS-170510-1
Purity	>99 %
Molecule weight	347,28 g/mol
Formula	$C_{11}H_{27}NO_7P_2$

Toxis Study Number SBW_10109182

2. Summary

The purpose of this study was to investigate the preliminary toxicity of the test item, 11-amino-1-hydroxyundecylidene-1,1,-bisphosphonic acid . Three different study types were used in order to investigate the test item acute toxicity:

1) Acute toxicity in rat:
The purpose of this study was to investigate the acute toxicity of the test item 11-amino-1-hydroxyundecylidene-1,1,-bisphosphonic acid, according to the OECD Fixed Dose procedure after single oral (p.o.) dose administered to rats.

2) AMES Test:
The bacteria reversed mutation assay (Ames Test) was used to evaluate the mutagenic properties of the test item with the five strain test option. The bacteria reversed mutation assay (Ames Test) was used to evaluate the mutagenic properties of the test item. This study was performed in full compliance with the OECD guidelines for testing of chemicals section 4, *Salmonella typhimurium* Reverse Mutation Assay, Test No.471 revised in December 1997.

3) Ecotoxicology
(a) Acute toxicity for *D. magna*: The purpose of acute toxicity test for *D. magna* test (SFS-EN ISO 6341; 1996) was to determine the median effective concentration for immobilization (EC50) of the test item to *D. magna* for 24 and 48 h.

(b) Biodegradability: Biodegradation potential of the test item was evaluated by a Chemical Oxygen Demand, COD(Cr) (ISO 15705; 2002).

3. Results

According to the results of the present study, the test item had no observed toxic effects in the acute toxicity study in rats, in the Ames-test or in the ecotoxicological experiments.
